# The Effects of poly-GA and poly-PR *C9orf72* Dipeptide Repeats on Sleep Patterns in *Drosophila melanogaster*

**DOI:** 10.17912/micropub.biology.000973

**Published:** 2024-03-01

**Authors:** Genevieve Uy, Laura N. Farrell, Syeda F. Faheem, Lauren E. Kinne, Madison G. Adore, Seol Hee Im, Robert Fairman

**Affiliations:** 1 Chemistry, Haverford College, Philadelphia, Pennsylvania, United States; 2 Neuroscience, Haverford College, Philadelphia, Pennsylvania, United States; 3 Biology, Haverford College, Philadelphia, Pennsylvania, United States

## Abstract

*C9orf72* is the most common familial gene associated with amyotrophic lateral sclerosis (ALS). Dipeptide repeats (DPRs) encoded by an expanded nucleotide repeat sequence in the *C9orf72* gene were found in the sleep-related neurons of patients, indicating a role of DPRs in ALS-associated sleep disruptions. Poly-GA or poly-PR DPRs were expressed in male *Drosophila melanogaster* to study their effect on sleep *.* Poly-PR expression caused sleep disruptions while poly-GA expression did not. This study validates the use of *Drosophila* as an *in vivo* model system for exploring the roles of DPRs in perturbing the underlying molecular mechanisms in sleep regulation.

**Figure 1. PR50 expression disrupts sleep in male flies, while GA50 expression has no effect:**
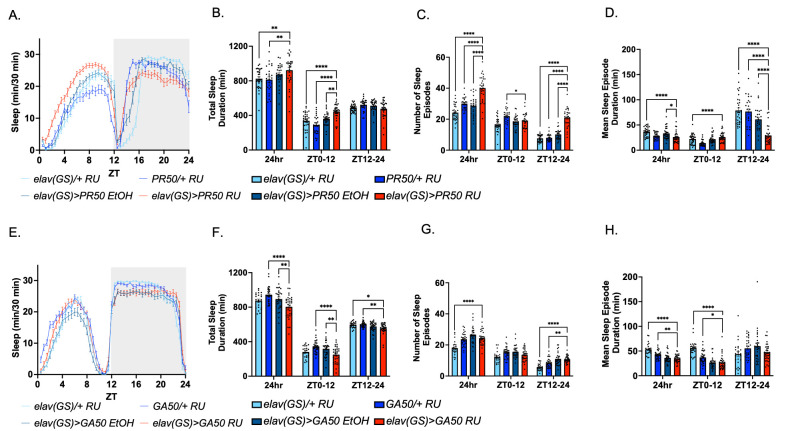
**Flies were treated with vehicle or RU486 for seven days and subsequently placed in the DAM tubes for three days of data collection.** These graphs are from one representative of three replicate experiments. (A-D) Sleep data collected from male fly sleep studies testing pan-neuronal PR50 expression averaged over three days: *elav(GS)/+* with RU486, n=32; *PR50/+* with RU486, n=26; *elav(GS)>PR50* vehicle control, n=29; *elav(GS)>PR50* with RU486, n=29; over 12hr:12hr light:dark cycles. (A) Sleep profile of flies expressing pan-neuronal PR50. (B) Total sleep duration in minutes. (C) Number of sleep episodes. (D) Mean sleep episode duration. (E-H) Sleep data collected from male fly sleep studies testing pan-neuronal GA50 expression averaged over three days: *elav(GS)/+* with RU486, n=24; *GA50/+* with RU486, n=32; *elav(GS)>GA50* vehicle control, n=29; *elav(GS)>GA50* with RU486, n=32; over 12hr:12hr light:dark cycles. (E) Sleep profile of flies expressing pan-neuronal GA50. (F) Total sleep duration in minutes. (G) Number of sleep episodes. (H), Mean sleep episode duration. * P ≤ 0.05; ** P ≤ 0.01; *** P<0.001; **** P ≤ 0.0001.

## Description

ALS is a motor neuron disease that causes patients to experience sleep problems like sleep fragmentation and excessive daytime sleepiness (Arnulf et al., 2000; Ting & Malhotra, 2005; Lo Coco et al., 2011; Boentert, 2020; Sun et al., 2020) . Recently, post-mortem staining of human brain tissue from patients with familial ALS revealed that cells associated with maintaining circadian rhythms, particularly pinealocytes and suprachiasmatic nucleus (SCN) neurons, showed dipeptide repeat (DPR) pathology, indicating a potential role of these DPRs in promoting sleep abnormalities (Dedeene et al., 2019) . DPRs arise from the translation of an abnormal expansion of the G _4_ C _2_ hexanucleotide repeat in the *C9orf72* gene (DeJesus-Hernandez et al., 2011; Majounie et al., 2012) . The translation of these hexanucleotide repeat expansions can generate five unique dipeptide repeats (DPRs) through the process of RAN translation: poly-GA, poly-PR, poly-GR, poly-GP, and poly-PA (Ash et al., 2013) . Of the DPRs, poly-GA and poly-PR have been the focus of many studies related to familial ALS. Poly-GA is the most abundant DPR generated in the disease (Lee et al., 2017) . Poly-GA aggregates into cytoplasmic inclusions that impair nucleocytoplasmic transport (NCT), which is an essential cellular mechanism that maintains sleep-related circadian rhythms (Zhang et al., 2016; Khosravi et al., 2017; Vanneste et al., 2019; Patke et al., 2020) . In contrast to poly-GA, poly-PR has low expression levels in cells, but has been shown to be amongst the most toxic of the DPRs (Mackenzie et al., 2015, Freibaum & Taylor, 2017) . Poly-PR has been shown to cause cellular toxicity through several mechanisms involved in maintaining circadian rhythms, including: nuclear localization and subsequent disruption of the transcription process, NCT disruption due to importin sequestration via liquid liquid phase separation (LLPS), and proteasome inhibition causing disrupted protein homeostasis (Wen et al., 2014; Boeynaems et al., 2016; Lee et al., 2016; Lin et al., 2016; Freibaum & Taylor, 2017; Gupta et al., 2017; White et al., 2019; Hayes et al., 2020; Hutten et al., 2020) . Despite the evidence of DPR expression in sleep-related brain cells, to our knowledge, no study so far has raised the question: does DPR expression affect sleep *in vivo* ? To answer this question, we expressed poly-PR and poly-GA DPRs in all neurons in male *D. melanogaster* , a well-established model for both sleep and the neurodegenerative effects of *C9orf72* DPRs (Cirelli & Bushey, 2008; Cirelli, 2009; Mizielinska et al., 2014) . In this study, we found that poly-PR expression disrupted fly sleep patterns while poly-GA did not, suggesting multiple modes of DPR-mediated toxicity.

We first focused on the effect of poly-PR expression on *D. melanogaster* sleep. We induced PR50 expression in all neurons of adult flies for seven days post-eclosion then recorded their daily activity levels for three days. The data presented here represent one of three repetitions of sleep experiments including control and experimental genotypes performed, with significance of the findings being identical across the repetitions. For the experimental group, elav(GS)>PR50 flies were administered RU486 to induce pan-neuronal PR50 expression. Genetic controls for the experimental group, elav(GS)/+ and PR50/+ flies treated with RU486, were included to control for the variance in sleep phenotypes that could arise from the genetic background. Work from other groups have shown that RU486 exposure does not have effects on sleep in such genetic control experiments (Wu et al., 2009; Afonso, et al., 2015) . Additionally, an RU486 vehicle control, where elav(GS)>PR50 flies were exposed to ethanol, was included to control for the effects of RU486 on sleep on the experimental genotype. Sleep data for the PR50-expressing groups were compared to all three controls to determine significant differences in sleep parameters. While the control flies had more sleep during the nighttime, PR50-expressing flies showed an increase in daytime sleep and decrease in nighttime sleep ( Fig. 1A ). This observation was explored in more detail in Figs. 1B-D. PR50-expressing flies had significantly more total sleep duration (TSD) during the ZT0-12 phase when compared to the three controls but did not have significantly different total sleep for ZT12-24 ( Fig. 1B ). Furthermore, PR50-expressing flies had an increased number of sleep episodes (NSE) during the ZT12-24 phase ( Fig. 1C ). Finally, PR50-expressing flies showed a lower mean sleep episode duration (MSED) during the ZT12-24 phase ( Fig. 1D ). Overall, these data suggest that PR50 expression caused sleep pattern changes in flies.

Similar to PR50, we induced GA50 expression in all neurons of adult flies for seven days post-eclosion then recorded their daily activity levels for three days. Three independent iterations of the sleep studies including control and experimental genotypes were carried out, consistent with the work described above for the PR50 flies. For the experimental group, elav(GS)>GA50 flies were administered RU486 to induce pan-neuronal GA50 expression. To rule out compounding variables of genetic background and RU486 treatment, we looked for significant differences between GA50-expressing flies compared to all three controls. Sleep profiles showed GA50-expressing flies had similar sleep patterns compared across all three controls ( Fig. 1E ). Furthermore, GA50-expressing flies had no significant differences in TSD, NSE, or MSED when compared across the three controls ( Fig. 1F, G, H ). Overall, these data suggest that GA50 expression does not change fly sleep patterns.

The effect of PR50 expression on sleep mimics the sleep fragmentation and excessive daytime sleepiness experienced by ALS patients (Arnulf et al., 2000; Ting & Malhotra, 2005; Lo Coco et al., 2011; Boentert, 2020; Sun et al., 2020) . PR50-expressing flies had an increase in the NSE and a decrease in the MSED in the ZT12-24 phase, indicating that flies were waking up more frequently and had shorter sleep duration. ALS patients also wake up frequently during the night, leading to shorter sleep periods. Additionally, PR50-expressing flies had increased TSD during the ZT0-12 phase, indicating that the flies slept more in the day compared to controls. ALS patients experience excessive daytime sleepiness as a result of fragmented nighttime sleep, resulting in an increased likelihood of falling asleep during the day. Our results of the PR50 sleep experiment suggest that PR50 may play a direct cellular role in affecting sleep.

In contrast to the PR50 expression, we noted that GA50 expression did not seem to have an effect on fly sleep parameters. GA50-expressing flies were compared to genetic controls and an ethanol vehicle control to account for effects of genetic background and RU486, respectively. The GA50-expressing group must be significantly different from all three controls in order to isolate the effect of GA50 expression on sleep parameters. Because the GA50-expressing group was not significantly different when compared to all three controls, we conclude that GA50 expression may not have an effect on sleep. These results suggest that, although poly-GA DPRs are the most highly expressed and have detrimental effects on brain health as described above, poly-GA DPRs may not have a direct effect on sleep. Alternatively, poly-GA may need to accumulate over time to cause sleep disruptions.

In this study, we presented a *Drosophila* model of ALS PR50 pathology that features sleep phenotypes similar to sleep problems experienced by ALS patients. Furthermore, we showed that GA50 expression did not affect *Drosophila* sleep. Our findings open the possibilities to future studies focused on determining the molecular basis of the effects of PR50 on sleep, as well as determining how other DPRs may contribute to sleep disruption.

## Methods

***Fly Husbandry***

*Drosophila* stocks and experimental crosses were maintained on standard *Drosophila* media at either 18°C or 25°C. The *elav(GS)-GAL4* driver was used to induce expression of GA50 and PR50 in progeny. Mifepristone (RU486, Sigma Aldrich) was added to standard media for a final concentration of 20 μg/mL. RU486 stocks were prepared in ethanol, aliquoted, and stored at -20°C. Adult male progeny from the experimental cross and genetic controls were collected 0-2 days post-eclosion then placed on RU486-containing media for 7 days. A vehicle control for the experimental cross was also performed using an equal amount of ethanol-containing media for 7 days. All progeny were entrained on a 12h:12h light:dark cycle prior to sleep experiments. All experimental work was carried out at 25°C in free-standing incubators.

***Drosophila Activity Monitoring and Sleep Analysis***

Male flies of the genetic and experimental groups were tested for sleep analysis using *Drosophila* Activity Monitors (DAMs, Trikinetics, Inc.) After 7-day RU486 or EtOH exposure, flies were placed individually into 65 mm glass tubes with 5% sucrose, 1% agarose (w/v) food medium **then immediately moved into a 25°C incubator with** 12h:12h light:dark conditions for 3-5 days of activity monitoring. Activity data were collected for each trial using the Trikinetics DAMSystem3 program (https://www.trikinetics.com/). Data for each trial were analyzed using the Vecsey Sleep and Circadian Analysis MATLAB Program (SCAMP). Graphs were produced using Excel and GraphPad Prism. Three repetitions of the sleep experiments were carried out to validate the results. One-way ANOVA was performed with Dunnett’s post-hoc when data had a normal distribution, otherwise a Kruskal-Wallis was performed with Dunn’s post-hoc test.

***Statistical Analysis Results***

Statistical analysis results for sleep experiments

**Table:** 

Figure Panel	Time Frame	Statistical Test	P Value	
B	24hr	Kruskal-Wallis	0.0041	
B	ZT0-12	ANOVA	<0.0001	
B	ZT12-24	Kruskal-Wallis	0.0150	
C	24hr	ANOVA	<0.0001	
C	ZT0-12	ANOVA	<0.0001	
C	ZT12-24	Kruskal-Wallis	<0.0001	
D	24hr	Kruskal-Wallis	0.0001	
D	ZT0-12	Kruskal-Wallis	<0.0001	
D	ZT12-24	Kruskal-Wallis	<0.0001	
F	24hr	ANOVA	<0.0001	
F	ZT0-12	ANOVA	<0.0001	
F	ZT12-24	Kruskal-Wallis	0.0049	
G	24hr	ANOVA	<0.0001	
G	ZT0-12	Kruskal-Wallis	0.0108	
G	ZT12-24	ANOVA	0.0029	
H	24hr	ANOVA	<0.0001	
H	ZT0-12	Kruskal-Wallis	<0.0001	
H	ZT12-24	Kruskal-Wallis	0.0648	

## Reagents

*Drosophila melanogaster* stocks used in experiments

**Table:** 

**Fly Lines**	**Genotype**	**Source**	**Identifier**
*UAS-FLAG-PR50-eGFP*	poly-PR50.UAS.Tag:FLAG,EGFP ^1^	Gift from Udai Pandey	FBtp0116650
*UAS-FLAG-GA50-eGFP*	poly-GA50.UAS.Tag:FLAG,EGFP ^1^	Gift from Udai Pandey	FBtp0116648
*elav(GS)-GAL4*	y[1] w[*]; P{w[+mC]=elav-Switch.O}GSG301	*Bloomington Drosophila Stock Center*	FBst0043642
* w ^1118^*	w[1118]	*Bloomington Drosophila Stock Center*	FBst0005905

^1^ Genotypes as reported in Flybase: https://flybase.org/reports/FBrf0228084

Reagents used

**Table:** 

**Reagents**	**Source**
*Mifepristone (RU486)*	Sigma Aldrich

## References

[R1] Afonso DJ, Liu D, Machado DR, Pan H, Jepson JE, Rogulja D, Koh K (2015). TARANIS Functions with Cyclin A and Cdk1 in a Novel Arousal Center to Control Sleep in Drosophila.. Curr Biol.

[R2] Arnulf I, Similowski T, Salachas F, Garma L, Mehiri S, Attali V, Behin-Bellhesen V, Meininger V, Derenne JP (2000). Sleep disorders and diaphragmatic function in patients with amyotrophic lateral sclerosis.. Am J Respir Crit Care Med.

[R3] Ash PE, Bieniek KF, Gendron TF, Caulfield T, Lin WL, Dejesus-Hernandez M, van Blitterswijk MM, Jansen-West K, Paul JW 3rd, Rademakers R, Boylan KB, Dickson DW, Petrucelli L (2013). Unconventional translation of C9ORF72 GGGGCC expansion generates insoluble polypeptides specific to c9FTD/ALS.. Neuron.

[R4] Boentert M (2020). Sleep and Sleep Disruption in Amyotrophic Lateral Sclerosis.. Curr Neurol Neurosci Rep.

[R5] Boeynaems S, Bogaert E, Michiels E, Gijselinck I, Sieben A, Jovičić A, De Baets G, Scheveneels W, Steyaert J, Cuijt I, Verstrepen KJ, Callaerts P, Rousseau F, Schymkowitz J, Cruts M, Van Broeckhoven C, Van Damme P, Gitler AD, Robberecht W, Van Den Bosch L (2016). Drosophila screen connects nuclear transport genes to DPR pathology in c9ALS/FTD.. Sci Rep.

[R6] Cirelli C (2009). The genetic and molecular regulation of sleep: from fruit flies to humans.. Nat Rev Neurosci.

[R7] Cirelli C, Bushey D (2008). Sleep and wakefulness in Drosophila melanogaster.. Ann N Y Acad Sci.

[R8] Dedeene L, Van Schoor E, Vandenberghe R, Van Damme P, Poesen K, Thal DR (2019). Circadian sleep/wake-associated cells show dipeptide repeat protein aggregates in C9orf72-related ALS and FTLD cases.. Acta Neuropathol Commun.

[R9] DeJesus-Hernandez M, Mackenzie IR, Boeve BF, Boxer AL, Baker M, Rutherford NJ, Nicholson AM, Finch NA, Flynn H, Adamson J, Kouri N, Wojtas A, Sengdy P, Hsiung GY, Karydas A, Seeley WW, Josephs KA, Coppola G, Geschwind DH, Wszolek ZK, Feldman H, Knopman DS, Petersen RC, Miller BL, Dickson DW, Boylan KB, Graff-Radford NR, Rademakers R (2011). Expanded GGGGCC hexanucleotide repeat in noncoding region of C9ORF72 causes chromosome 9p-linked FTD and ALS.. Neuron.

[R10] Freibaum, B. D., & Taylor, J. P. (2017). The Role of Dipeptide Repeats in C9ORF72-Related ALS-FTD. *Frontiers in Molecular Neuroscience* , *10* , 35. 10.3389/fnmol.2017.00035 PMC5303742 28243191

[R11] Gupta R, Lan M, Mojsilovic-Petrovic J, Choi WH, Safren N, Barmada S, Lee MJ, Kalb R (2017). The Proline/Arginine Dipeptide from Hexanucleotide Repeat Expanded
*C9ORF72*
Inhibits the Proteasome.. eNeuro.

[R12] Hayes LR, Duan L, Bowen K, Kalab P, Rothstein JD (2020). C9orf72 arginine-rich dipeptide repeat proteins disrupt karyopherin-mediated nuclear import.. Elife.

[R13] Hutten S, Usluer S, Bourgeois B, Simonetti F, Odeh HM, Fare CM, Czuppa M, Hruska-Plochan M, Hofweber M, Polymenidou M, Shorter J, Edbauer D, Madl T, Dormann D (2020). Nuclear Import Receptors Directly Bind to Arginine-Rich Dipeptide Repeat Proteins and Suppress Their Pathological Interactions.. Cell Rep.

[R14] Khosravi B, Hartmann H, May S, Möhl C, Ederle H, Michaelsen M, Schludi MH, Dormann D, Edbauer D (2017). Cytoplasmic poly-GA aggregates impair nuclear import of TDP-43 in C9orf72 ALS/FTLD.. Hum Mol Genet.

[R15] Lee KH, Zhang P, Kim HJ, Mitrea DM, Sarkar M, Freibaum BD, Cika J, Coughlin M, Messing J, Molliex A, Maxwell BA, Kim NC, Temirov J, Moore J, Kolaitis RM, Shaw TI, Bai B, Peng J, Kriwacki RW, Taylor JP (2016). C9orf72 Dipeptide Repeats Impair the Assembly, Dynamics, and Function of Membrane-Less Organelles.. Cell.

[R16] Lee YB, Baskaran P, Gomez-Deza J, Chen HJ, Nishimura AL, Smith BN, Troakes C, Adachi Y, Stepto A, Petrucelli L, Gallo JM, Hirth F, Rogelj B, Guthrie S, Shaw CE (2017). C9orf72 poly GA RAN-translated protein plays a key role in amyotrophic lateral sclerosis via aggregation and toxicity.. Hum Mol Genet.

[R17] Lin Y, Mori E, Kato M, Xiang S, Wu L, Kwon I, McKnight SL (2016). Toxic PR Poly-Dipeptides Encoded by the C9orf72 Repeat Expansion Target LC Domain Polymers.. Cell.

[R18] Lo Coco D, Mattaliano P, Spataro R, Mattaliano A, La Bella V (2011). Sleep-wake disturbances in patients with amyotrophic lateral sclerosis.. J Neurol Neurosurg Psychiatry.

[R19] Mackenzie IR, Frick P, Grässer FA, Gendron TF, Petrucelli L, Cashman NR, Edbauer D, Kremmer E, Prudlo J, Troost D, Neumann M (2015). Quantitative analysis and clinico-pathological correlations of different dipeptide repeat protein pathologies in C9ORF72 mutation carriers.. Acta Neuropathol.

[R20] Majounie E, Renton AE, Mok K, Dopper EG, Waite A, Rollinson S, Chiò A, Restagno G, Nicolaou N, Simon-Sanchez J, van Swieten JC, Abramzon Y, Johnson JO, Sendtner M, Pamphlett R, Orrell RW, Mead S, Sidle KC, Houlden H, Rohrer JD, Morrison KE, Pall H, Talbot K, Ansorge O, Hernandez DG, Arepalli S, Sabatelli M, Mora G, Corbo M, Giannini F, Calvo A, Englund E, Borghero G, Floris GL, Remes AM, Laaksovirta H, McCluskey L, Trojanowski JQ, Van Deerlin VM, Schellenberg GD, Nalls MA, Drory VE, Lu CS, Yeh TH, Ishiura H, Takahashi Y, Tsuji S, Le Ber I, Brice A, Drepper C, Williams N, Kirby J, Shaw P, Hardy J, Tienari PJ, Heutink P, Morris HR, Pickering-Brown S, Traynor BJ, Chromosome 9-ALS/FTD Consortium, French research network on FTLD/FTLD/ALS, ITALSGEN Consortium (2012). Frequency of the C9orf72 hexanucleotide repeat expansion in patients with amyotrophic lateral sclerosis and frontotemporal dementia: a cross-sectional study.. Lancet Neurol.

[R21] Mizielinska S, Grönke S, Niccoli T, Ridler CE, Clayton EL, Devoy A, Moens T, Norona FE, Woollacott IOC, Pietrzyk J, Cleverley K, Nicoll AJ, Pickering-Brown S, Dols J, Cabecinha M, Hendrich O, Fratta P, Fisher EMC, Partridge L, Isaacs AM (2014). C9orf72 repeat expansions cause neurodegeneration in Drosophila through arginine-rich proteins.. Science.

[R22] Patke, A., Young, M. W., & Axelrod, S. (2020). Molecular mechanisms and physiological importance of circadian rhythms. *Nature Reviews Molecular Cell Biology* , *21* (2), 67–84. PMID: 31768006 10.1038/s41580-019-0179-2 31768006

[R23] Sun X, Zhao X, Liu Q, Liu S, Zhang K, Wang ZL, Yang X, Shang L, Huang Y, Cui L, Zhang X (2020). Study on sleep-wake disorders in patients with genetic and non-genetic amyotrophic lateral sclerosis.. J Neurol Neurosurg Psychiatry.

[R24] Ting L, Malhotra A (2005). Disorders of sleep: an overview.. Prim Care.

[R25] Vanneste J, Vercruysse T, Boeynaems S, Sicart A, Van Damme P, Daelemans D, Van Den Bosch L (2019). C9orf72-generated poly-GR and poly-PR do not directly interfere with nucleocytoplasmic transport.. Sci Rep.

[R26] Wen X, Tan W, Westergard T, Krishnamurthy K, Markandaiah SS, Shi Y, Lin S, Shneider NA, Monaghan J, Pandey UB, Pasinelli P, Ichida JK, Trotti D (2014). Antisense proline-arginine RAN dipeptides linked to C9ORF72-ALS/FTD form toxic nuclear aggregates that initiate in vitro and in vivo neuronal death.. Neuron.

[R27] White MR, Mitrea DM, Zhang P, Stanley CB, Cassidy DE, Nourse A, Phillips AH, Tolbert M, Taylor JP, Kriwacki RW (2019). C9orf72 Poly(PR) Dipeptide Repeats Disturb Biomolecular Phase Separation and Disrupt Nucleolar Function.. Mol Cell.

[R28] Wu MN, Ho K, Crocker A, Yue Z, Koh K, Sehgal A (2009). The effects of caffeine on sleep in Drosophila require PKA activity, but not the adenosine receptor.. J Neurosci.

[R29] Zhang YJ, Gendron TF, Grima JC, Sasaguri H, Jansen-West K, Xu YF, Katzman RB, Gass J, Murray ME, Shinohara M, Lin WL, Garrett A, Stankowski JN, Daughrity L, Tong J, Perkerson EA, Yue M, Chew J, Castanedes-Casey M, Kurti A, Wang ZS, Liesinger AM, Baker JD, Jiang J, Lagier-Tourenne C, Edbauer D, Cleveland DW, Rademakers R, Boylan KB, Bu G, Link CD, Dickey CA, Rothstein JD, Dickson DW, Fryer JD, Petrucelli L (2016). C9ORF72 poly(GA) aggregates sequester and impair HR23 and nucleocytoplasmic transport proteins.. Nat Neurosci.

